# SARS-CoV-2 induced IgA vasculitis confirmed with SARS-CoV-2 tissue testing

**DOI:** 10.1016/j.jdcr.2023.11.010

**Published:** 2023-11-25

**Authors:** Ahmed N. Ansari, Emma F. Johnson, Katherine L. Wang, Matthew J. Koster, Hafsa M. Cantwell

**Affiliations:** a Department of Dermatology, Mayo Clinic, Rochester, Minnesota; b Department of Laboratory Medicine and Pathology, Mayo Clinic, Rochester, Minnesota; c Mayo Clinic Alix School of Medicine, Mayo Clinic, Jacksonville, Florida; d Department of Rheumatology, Mayo Clinic, Rochester, Minnesota

**Keywords:** COVID-19, COVID-19 testing, IgA vasculitis, polymerase chain reaction, SARS-CoV-2

## Introduction

A wide range of cutaneous morphologies have been described in association with severe acute respiratory syndrome coronavirus 2 (SARS-CoV-2) infection, the most common being morbilliform, pernio-like, and urticarial lesions.^1^^,^^2^ Histologically confirmed cutaneous vasculitis and Kawasaki-like vasculitis have also been described in association with COVID-19.^1^, ^2^, ^3^ Here, we present a unique case of SARS-CoV-2 induced IgA vasculitis confirmed with SARS-CoV-2 tissue testing on biopsy.

## Case report

A 50-year-old male with a history of gout on long-term allopurinol presented for acute-onset pruritic lower extremity rash. The rash began 1 week before presentation, diffusely involving the bilateral lower extremities and abdomen. He developed diarrhea after rash onset. Review of systems was otherwise negative. He was unvaccinated for COVID-19. Treatments including compression and an over-the-counter antibiotic ointment were trialed without improvement. He denied any new medications.

**Fig 1:**
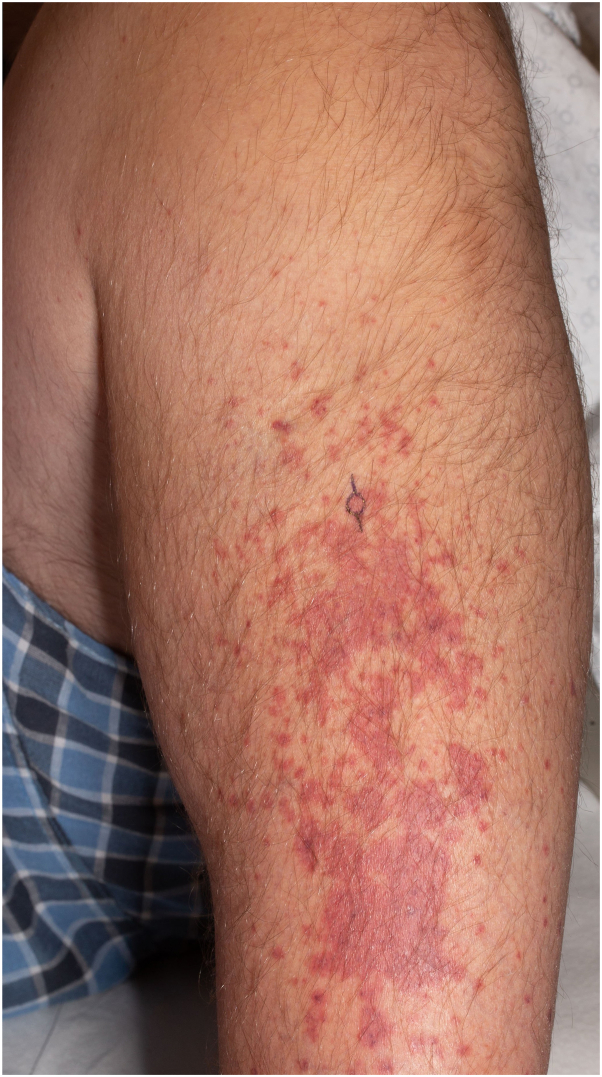
Physical examination revealed multiple coalescing pink macules and papules along with palpable purpura on the bilateral lower extremities.

**Fig 2:**
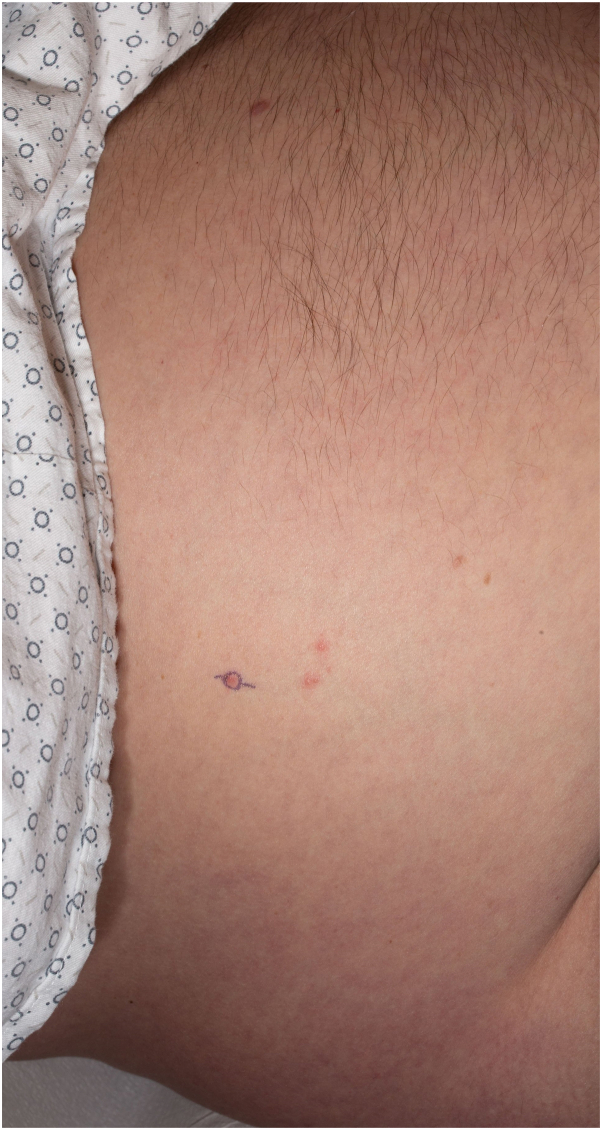
Examination revealed scattered pink papules and macular purpura on the abdomen.

Physical examination revealed coalescing macular and targetoid purpura plus multiple pink papules and palpable purpura on the bilateral lower extremities (Fig 1) as well as scattered pink papules and macular purpura on the abdomen (Fig 2). Bilateral ankle swelling was noted.

Comprehensive laboratory evaluation was unrevealing except for elevated antistreptolysin O titer and C-reactive protein and borderline elevated total complement (Supplementary Table I, available via Mendeley at https://data.mendeley.com/datasets/pzhrj9rd36/1). COVID-19 polymerase chain reaction (PCR) nasal swab test was negative. Computed tomography of the chest, abdomen, and pelvis with angiography was unremarkable.

**Fig 3:**
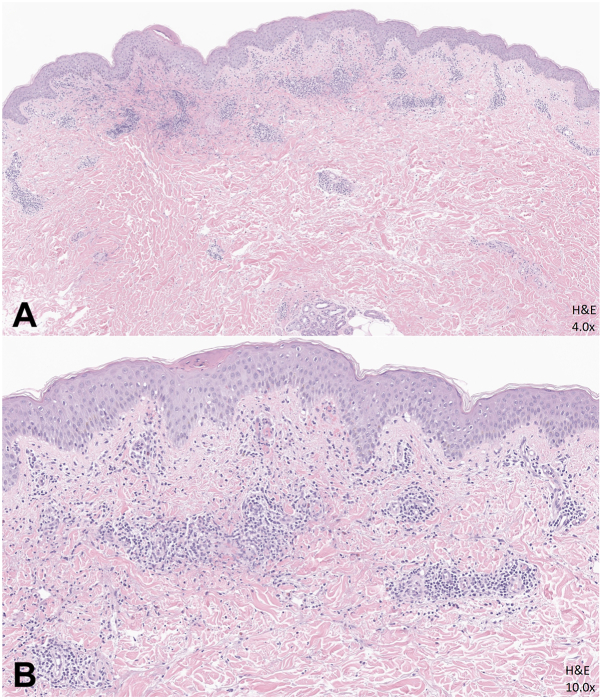
A 6-mm punch biopsy from the right upper abdomen demonstrated leukocytoclastic vasculitis on hematoxylin and eosin evaluation. Panel **(A)** shows ×4 magnification and panel **(B)** shows ×10 magnification. SARS-Co-V-2 RNA was detected in this tissue sample.

**Fig 4:**
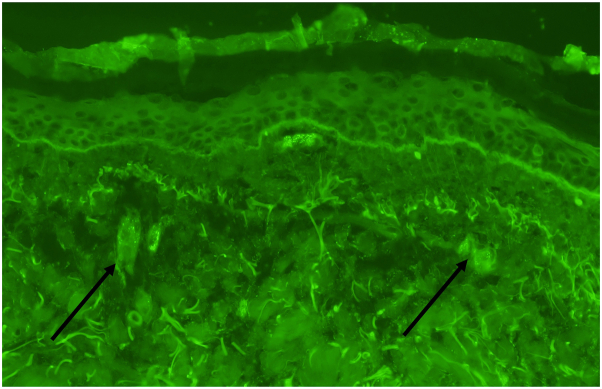
Representative image of direct immunofluorescence (DIF) demonstrating granular IgA deposition (*arrows*) within the walls of dermal vessels.

Punch biopsies from affected sites demonstrated leukocytoclastic vasculitis on hematoxylin and eosin (Fig 3). Direct immunofluorescence showed granular IgA deposition within the walls of many superficial dermal vessels (Fig 4). SARS-CoV-2 staining was ordered due to patient report of new-onset diarrhea and his unvaccinated status for further investigation of infectious etiology. SARS-CoV-2 RNA droplet digital PCR (ddPCR) performed on the formalin-fixed, paraffin-embedded tissue from the abdomen was positive.

The patient was diagnosed with IgA vasculitis secondary to otherwise asymptomatic COVID-19 as the inciting factor. He did not have any recent respiratory symptoms, which made streptococcus infection unlikely. Allopurinol was the only potential culprit medication; however, he had been on allopurinol for several years without concerns. His vasculitis was successfully treated with prednisone 40 mg daily with 4-week taper and midpotency topical corticosteroids. Of note, the patient did have a positive SARS-CoV-2 RNA PCR swab test 2 months after his initial presentation, and he was treated with monoclonal antibody infusion for progressive respiratory symptoms. He has now had resolution of his cutaneous vasculitis and recovered from his COVID-19 as well.

## Discussion

The COVID-19 pandemic is continuously evolving, and practitioners should be aware of the variety of associated cutaneous and systemic manifestations. Dermatologic morphologies include morbilliform, pernio-like, urticarial, papulosquamous, macular erythematous, vesicular, and retiform purpura.^2^ Although SARS-CoV-2 vaccination-induced cutaneous vasculitis has been reported, small-vessel vasculitis during early or active SARS-CoV-2 is rare.^4^ Clinicians should have a high index of suspicion when evaluating vasculitis. Our case highlights the importance of considering COVID-19 induced vasculitis as a potential etiology.

This case emphasizes the utility of COVID-19 testing on tissue as a method of diagnosing COVID-19 in the setting of a negative SARS-CoV-2 PCR nasal swab in patients with clinical suspicion for COVID-19 due to risk factors or symptoms or idiopathic new-onset cutaneous vasculitis. Colmenero et al^5^ described 7 patients with COVID-19 chilblains, with 6 tested and having negative SARS-CoV-2 PCR swabs but all 7 having immunohistochemical positivity for the SARS-CoV-2 spike protein.^5^ Thus, COVID-19 testing on tissue can provide a causal relation of cutaneous lesions with SARS-CoV-2 and add to the spectrum of dermatologic manifestations of SARS-CoV-2 infection.^5^^,^^6^

Droplet digital PCR (ddPCR) was used in this case as a confirmatory test after a negative nasal swab PCR. Compared with standard nasal swab PCR, ddPCR demonstrates higher sensitivity in detecting samples with low SARS-CoV-2 viral load, resulting in a lower rate of false negative results.^7^ Therefore, ddPCR may be considered as an adjunct test when clinical suspicion remains high in the setting of a negative nasal swab. Limitations of ddPCR include lack of automation, difficulty screening numerous samples, and higher cost when compared with standard nasal swab PCR.^8^ Renal sequelae are common in adults with IgA vasculitis, with up to 76% of adult patients reported to have hematuria, proteinuria, or red blood cell casts in a review by Yaseen et al.^9^ The patient had unremarkable urinalysis and blood pressure monitoring during the course of his illness and at his most recent follow-up visit 2 years after his initial presentation.

Cutaneous manifestations of COVID-19 typically present simultaneously with or after other COVID-19 symptoms; however, in 12% of cases, they can occur prior.^2^ Diagnosing underlying COVID-19 is paramount, given the associated public health risks, since accurate and timely diagnosis can prevent further spread of the virus through appropriate quarantining measures and possibly provide patients with COVID-19 treatment options earlier in their disease course.

## Conflicts of interest

None disclosed.
